# Genome-wide identification for genes involved in sodium dodecyl sulfate toxicity in *Saccharomyces cerevisiae*

**DOI:** 10.1186/s12866-020-1721-2

**Published:** 2020-02-17

**Authors:** Chunlei Cao, Zhengfeng Cao, Peibin Yu, Yunying Zhao

**Affiliations:** 1 National Engineering Laboratory for Cereal Fermentation Technology (NELCF), Jiangnan University, 1800 Lihu Road, Wuxi, 214122 Jiangsu China; 2 Jiangsu Provincial Research Center for Bioactive Product Processing Technology, Jiangnan University, 1800 Lihu Road, Wuxi, 214122 Jiangsu China; 3 College of Animal Science and Technology, Yangzhou University, Yangzhou, 225009 Jiangsu China

**Keywords:** *Saccharomyces cerevisiae*, SDS, Genetic screening, Genomics, ROS

## Abstract

**Background:**

Sodium dodecyl sulfate (SDS) is one of the most widely used anionic alkyl sulfate surfactants. Toxicological information on SDS is accumulating, however, mechanisms of SDS toxicity regulation remain poorly understood. In this study, the relationship between the SDS-sensitive mutants and their intracellular ROS levels has been investigated.

**Results:**

Through a genome-scale screen, we have identified 108 yeast single-gene deletion mutants that are sensitive to 0.03% SDS. These genes were predominantly related to the cellular processes of metabolism, cell cycle and DNA processing, cellular transport, transport facilities and transport routes, transcription and the protein with binding function or cofactor requirement (structural or catalytic). Measurement of the intracellular ROS (reactive oxygen species) levels of these SDS-sensitive mutants showed that about 79% of SDS-sensitive mutants accumulated significantly higher intracellular ROS levels than the wild-type cells under SDS stress. Moreover, SDS could generate oxidative damage and up-regulate several antioxidant defenses genes, and some of the SDS-sensitive genes were involved in this process.

**Conclusion:**

This study provides insight on yeast genes involved in SDS tolerance and the elevated intracellular ROS caused by SDS stress, which is a potential way to understand the detoxification mechanisms of SDS by yeast cells.

## Background

Surfactants are organic pollutants distributed widely in the current environment, and their toxicity has caused widespread concern. One of the synthetic anionic surfactants, sodium dodecyl sulfate (SDS), or sodium lauryl sulfate (SLS), a product that consists of approximately 70% sodium dodecyl sulfate and 30% sodium tetradecyl sulfate, with the formula of CH3(CH2)_11_OSO_3_Na, has been used in many cleaning and hygiene products such as liquid soaps, shampoos, bubble baths, shower gels, and nearly all toothpastes. SDS is also used in pharmaceutical and food products, as well as in industiral and laboratory applications, i.e., SDS can form complexes with protein through hydrophobic interactions and thus be used in polyacrylamide gel electrophoresis to determine the molecular weight of proteins [1, 2]. The concentration of SDS found in consumer products varies by product and manufacturer but typically ranges from 0.01 to 50% in cosmetic products and 1 to 30% in cleaning products [3]. The lethal dose, 50% (LD50) for SDS is 0.8–1.10 g/kg in rats, SDS concentrations >2% are considered irritating to normal skin in human patch testing, and > 5% causes depression, labored breathing, diarrhea, and death (four out of 20 animals) [2].

Safety concerns with SDS application in human include carcinogenicity, skin and eye irritation, and aphthous ulcers. The toxicity of SDS has been demonstrated in bacteria, microalgae, crustaceans, echinoderms, rats, humans and carp. The basis of SDS toxicity seems to be mainly related to the alteration of the cellular ionic balance caused by cellular membrane permeability alterations and to the induction of oxidative stress, that can generate other physiological and biochemical stresses [4]. SDS elicits both physical and biochemical effects on cells, with the membrane the primary target structure, and considered as a a typical cell wall perturbing agent. Effects are concentration dependent and range from loss of barrier function and increased permeability to complete cell lysis. It is suggested that SDS causes elevated the glutathione production, lipid peroxidation as well as changes in carbon metabolism [5], leading to altered cell membrane stability and permeability as well as indirectly to increased accessibility of cell wall [6]. Yeast cell wall serves crucial functions in protecting against osmotic shock stress and mechanical steess, maintaining cell shape, as well as serving a sacffold for cell-surface proteins [7]. SDS interrupts cell membranes and then triggers the Cell Wall Integrity (CWI) signaling pathway, a kinase cascade to maintain cell integrity and can be activated by chemicals that damage the cell wall and membrane in buding yeast [8]. For example, the Slt2/Mpk1, a mitogen-activated protein (MAP) kinase, can be phosphorylated and thus activated by impaired cell integrity [9]. However, deatiled mechanisms of SDS toxicity in microorganisms or higher eukaryotes are poorly understood.

Yeast has been previously used to demonstrate the effect of SDS on biological membranes, showing that micelles of SDS may penetrate the membrane through pores in the yeast cell wall and destroy the membrane [10]. In defense against SDS surplus, yeast cells increase the expression levels of genes involved in oxidative stress which might be caused by its effect on membrane structure, carbon metabolism, or DNA repair [2]. Reactive oxygen spesies (ROS) play an important role in inducing cell death or apotosis in yeast cells by causing damages to proteins, lipids and DNA [11, 12]. In addtion, ROS could induce cell wall damage in yeast cells lacking mitochondrial DNA, making cells to become more sensitive to of SDS stress [13].

As the simplest eukaryotic organism, the budding yeast *Saccharomyces cerevisiae (S. cerevisiae)* has been used to identify the mechanism and regulation of metal ion transport [14]. Here, we used *S. cerevisiae* to explore the SDS effect on eukaryotic cell growth and compared the oxidative stress (reactive oxygen species, ROS) in cultured cells. We have firstly screened the SDS-sensitive mutants from the yeast nonessential gene deletion library and identified 108 SDS-sensitive mutants. To evaluate whether SDS generates serious oxidative stress to the SDS-sensitive mutant cells, we have then measured the cellular response of cultured yeast cells to SDS in terms of ROS levels. Specifically, we show that SDS can induce oxidative stress and that yeast cells eliminate these oxidative damage by elevating the expression levels of the genes coding for antioxidant defenses.

## Results

### An overview of genes involved in the SDS sensitivity of yeast cells

**Fig. 1:**
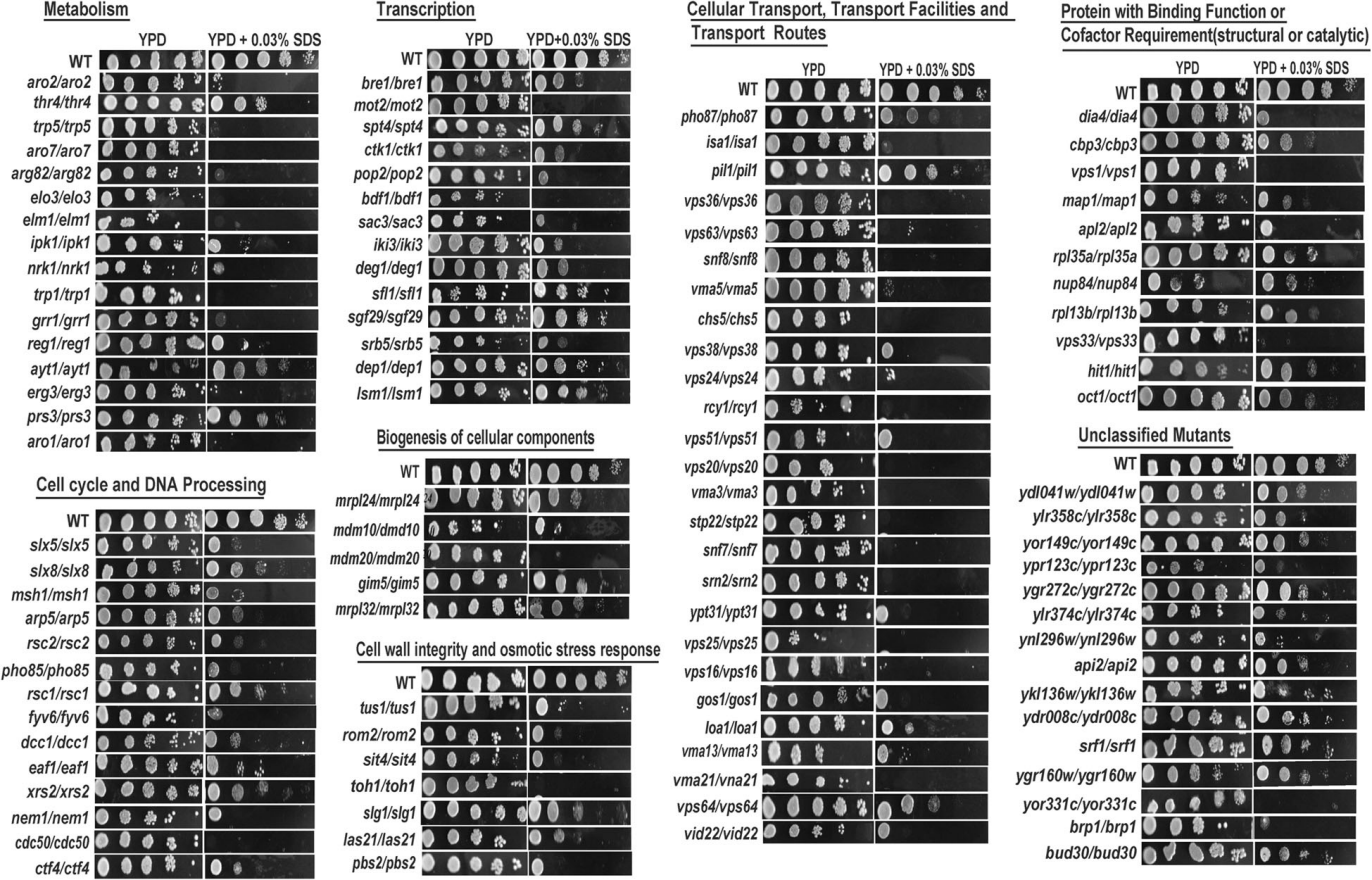
Phenotypes of the 108 SDS-sensitive deletion mutants of each functional category. Cells of the wild-type BY4743 and SDS-sensitive gene deletion mutants identified from the genome-scale screen were grown at 30 °C in liquid YPD overnight, serially diluted by 10 times and spotted on YPD plates with or without 0.03% SDS, respectively. Plates were incubated for 2 days at 30 °C. SDS, sodium dodecyl sulfate

To investigate the cellular functions required for cell growth under a surplus of SDS, a yeast library of diploid nonessential gene deletion was screened to identify genes involved in the sensitivity to SDS. The results show that 108 gene deletion mutants (2.3% of the screened 4757 gene deletion mutants) were identified as sensitive to 0.03% SDS (Fig. 1 and Table 1). The genotypes of these 108 mutants were confirmed by PCR with the forward primer derived from the promoter region of each correspondent gene and a reverse primer KanMX4-R (Additional file 1: Table S1 and Additional file 2: Fig. S1) derived from the ORF region of KanMX4. The functional categories of these 108 genes are involved in metabolism (17), cell cycle and DNA processing (15), transcription (14), cellular transport, transport facilities and transport routes (28), biogenesis of cellular components (6), cellular communication / signal transduction mechanism (2), protein with binding function or cofactor requirement (structural or catalytic) (10), as well as unclassified proteins (16) (Table 1). Gene Ontology (GO) enrichment analysis result showed that these 108 SDS-sensitive genes were mainly enriched in vacuolar transport, ATP export, and endosomal transport among the top 16 GO terms in cluster groups (Additional file 3: Fig. S2).

**Table 1:** Functional categories of 108 genes whose deletion mutants are sensitive to 0.03% SDS

Function	Genes							
Metabolism (16)	PRS3^*^	THR4^**^	ARG82^****^	REG1^***^	IPK1^***^	ARO1^*****^	TRP1^*****^	TRP5^*****^
	ELM1^*****^	AYT1^*^	ELO3^*****^	ARO7^*****^	ARO2^****^	NRK1^****^	GRR1^****^	ERG3^****^
Cell cycle and DNA processing (14)	NEM1^****^	CDC50^*****^	SLX5^***^	PHO85^***^	MSH1^***^	DCC1^***^	EAF1^**^	*XRS2*
	RSC2^***^	FYV6^****^	ARP5^**^	CTF4^**^	RSC1^*^	SLX8^*^		
Transcription (14)	BRE1^**^	BDF1^*****^	DEP1^*^	SAC3^****^	MOT2^*****^	DEG1^***^	SFL1^*^	POP2^****^
	CTK1^***^	LSM1^*^	SRB5^***^	SPT4^*^	IKI3^***^	SGF29^*^		
Cellular transport, transport facilities and transport routes (28)	GOS1^****^	PHO87^**^	VPS23^*****^	VMA3^*****^	YPT31^****^	LOA1^***^	VMA13^****^	VPS22^*****^
	VPS36^*****^	VPS38^****^	VPS37^*****^	VPS63^*****^	VPS24^****^	VPS25^*****^	RCY1^*****^	VPS51^****^
	CHS5^*****^	VPS32^*****^	ISA1^****^	PIL1^**^	VMA5^****^	VPS20^*****^	VPS16^*****^	VPS1^*****^
	VPS33^*****^	VPS64^**^	VMA21^*****^	VID22^****^				
Protein with binding function or cofactor requirement (structural or catalytic) (9)	DIA4^****^	RPL35A^**^	NUP84^**^	APL2^****^	MAP 1^**^	RPL13B^**^	CBP3^**^	HIT1^*^
	OCT1^**^							
Biogenesis of cellular components (5)	MDM10^***^	MRPL32^*^	MRPL24^**^	GIM5^*^	MDM20^*****^			
Cell wall integrity and osmotic stress response (7)	TUS1^***^	ROM2^***^	SIT4^***^	TOH1^*****^	SLG1^*^	LAS21^**^	PBS2^****^	
Unclassified proteins (15)	SRF1^*^	YDL041W^*^	YLR358C^**^	YNL296W^***^	YPR123C^***^	YDR149C^*^	YOR331C^*****^	YGR272C^*^
	YLR374C^*^	YKL136W^*^	YGR160W^*^	BRP1^****^	BUD30^*^	API2^**^	YDR008C^*^	

The number of asterisks represents SDS-sensitivity of different mutants. Mutant with five asterisks was most sensitive to SDS stress, while mutant with one asterisk was least sensitive to SDS

### Exposure to SDS stress results in ROS generation

**Fig. 2:**
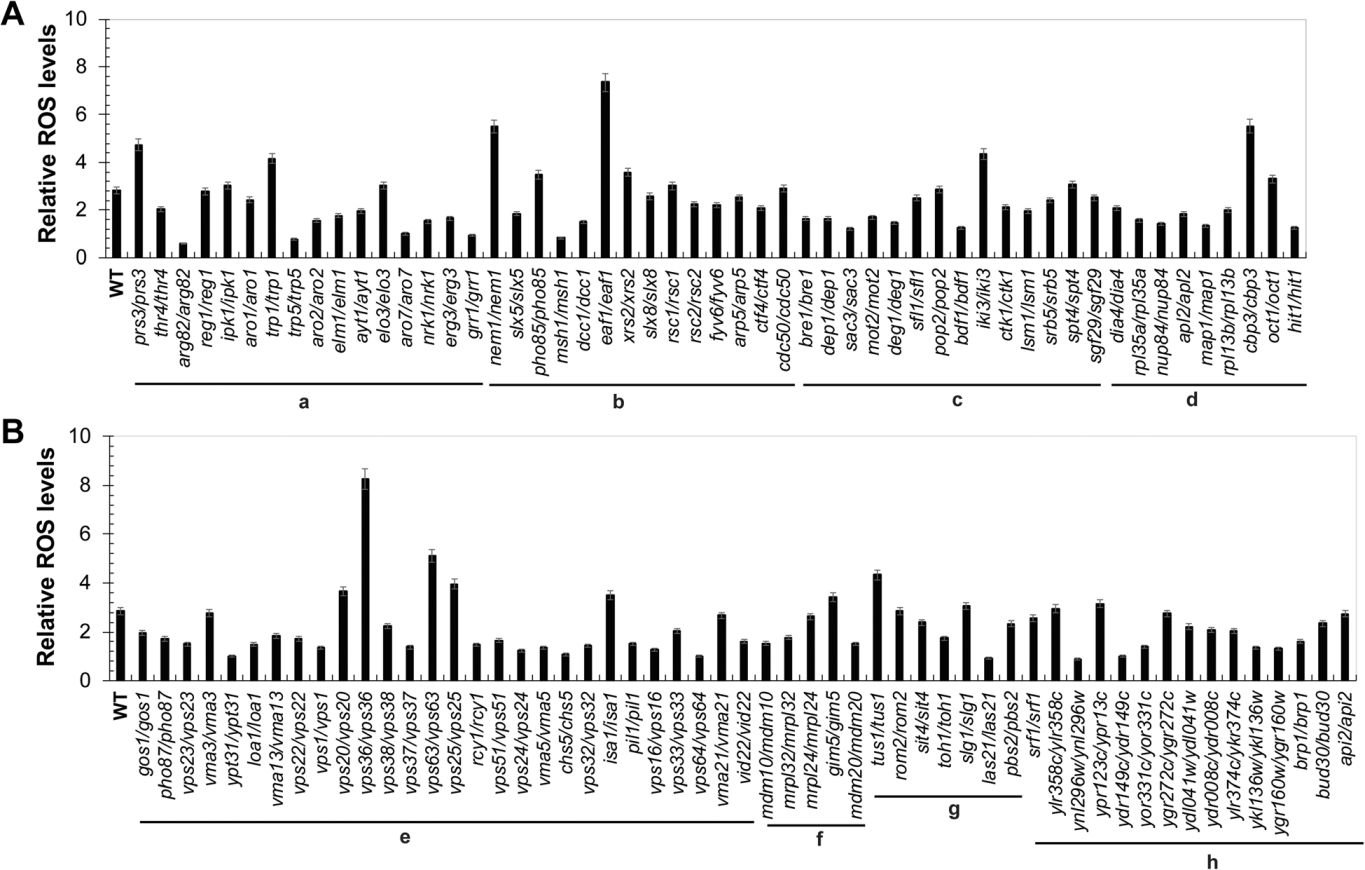
Relative ROS levels of 108 SDS-sensitive gene mutants in response to SDS stress. **a**: Metabolism; **b**: Cell cycle and DNA Processing; **c**: Transcription; **d**: Protein with Binding Function or Cofactor Requirement (structural or catalytic); **e**: Cellular Transport, Transport Facilities and Transport Routes; **f**: Biogenesis of cellular components; **g**: Cell wall integrity and osmotic stress respons; **h**: Unclassified Proteins. Log-phase cells were grown with or without 0.015% SDS for 2 h before they were collected for measurement of intracellular ROS levels stained by the dihydroethidium. The relative ROS levels of these SDS-sensitive mutants were listed according to their categories. Each date indicated the ratio between levels of ROS in YPD + SDS versus YPD alone. The value is the average of three independent assays for each strain

Since SDS had been confirmed to induce the oxidative stress response [2], we next measured the intracellular ROS levels of the 108 SDS-sensitive mutants under 0.015% SDS treatment. In the wild-type BY4743 cells, the intracellular ROS level was significantly increased under SDS stress (Fig. 2 and Additional file 4: Fig. S3). Interestingly, only six mutants for *ARG82*, *TRP5*, *GRR1*, *MSH1*, *LAS21*, and *YNL296W* of these 108 SDS-sensitive mutants, accumulated lower intracellular ROS levels when treated with 0.015% SDS than without SDS (The relative ROS levels in these mutants was smaller than 1; Fig. 2 and Additional file 4: Fig. S3). It suggested that the above six genes might not be directly involved in the regulation of intracellular ROS levels under SDS stress. Of these 108 SDS-sensitive mutants, 85 mutants accumulated significantly higher intracellular ROS levels under SDS stress compared with wild-type cells (Additional file 4: Fig. S3B and D), indicating that these 85 mutants might respond to lower concentration of SDS and thereby accumulated higher ROS levels than wild type cells. The rest 23 mutants accumulated similar or lower intracellular ROS levels when treated with SDS compared with wild type cells, although the relative ROS levels in some of these mutants were also very high (Fig.2 and Additional file 4: Fig. S3B and D). Here we showed that mutants for genes related to the functions of metabolism and cellular transport, transport facilities and transport routes were most sensitive to SDS stress (Table 1). We listed some genes as the representative genes of their categories as below.

### Genes involved in cellular transport and transport routes are associated with SDS tolerance

The largest functional category of these 108 identified SDS-sensitive genes is the cellular transport, transport facilities and transport routes (Table 1), including 28 genes identified. There are 63 nonessential vacuolar protein sorting (VPS) genes in the genome of *S. cerevisiae* [15]. Notably, 15 mutants for *VPS1*, *VPS16*, *VPS20, VPS24, VPS22, VPS23, VPS25, VPS32, VPS33, VPS36, VPS37,VPS38, VPS51, VPS63*, and *VPS64* were identified being sensitive to 0.03% SDS in the present study (Table 1; Fig. 1). The intracellular ROS levels of these 15 mutants were all induced by SDS stress, especially in mutants for *VPS20*, *VPS36*, *VPS63* and *VPS25* (Fig. 2). The results suggest that the VPS pathway involved in protein trafficking and membrane fusion plays an important role in the response of yeast cells to SDS stress.

The H^+^-ATPase localized in the membrane of vacuole (V-ATPase) is composed of the catalytic V1 subcomplex and the proton-translocating membrane V0 subcomplex, playing crucial roles in the organelles acidification and other intracellular activities [16, 17]. In this study, four mutants for *VMA3*, *VMA5*, *VMA13*, and *VMA21* were sensitive to 0.03% SDS (Table 1; Fig. 1). *VMA5* and *VMA13* encodes the V1 complex subunit C and H [18, 19], respectively. *VMA3* encodes the subunit c of the V0 complex [20]. *VMA21* is not an actual component of the V-ATPase complex, but encodes proteins functioned in the assembly of the V-ATPase [21]. These results indicate that the V-ATPase is critical for *S. cerevisiae* cells in responding to SDS in the environment.

### Mutants for genes involved in cell cycle and DNA processing render yeast cells sensitive to SDS stress

There are 14 genes identified in our study that are involved in cell cycle and DNA processing (Table 1; Fig. 1). The intracellular ROS levels in 13 mutants except the mutants for *MSH1* were all increased under SDS stress, especially in mutants for *MEM1*, *PHO85*, *EAF1* and *XRS2* (Fig. 2). *SLX5* and *SLX8* encode the subunit of Slx5-Slx8 ubiquitin-like modifier (SUMO)-targeted ubiquitin ligase (STUbL) complex [22–24]. Mutants for *SLX5* or *SLX8* were sensitive to 0.03% SDS (Table 1 and Fig. 1), suggesting that STUbL complex is involved in SDS tolerance of yeast cells. The small SUMO-targeted ubiquitin ligase complex is a nuclear ubiquitin ligase complex that specifically targets sumoylated proteins. It is formed of homodimers or heterodimers of RING finger protein 4 family ubiquitin ligases and is conserved in eukaryotes [23]. Three genes, *MSH1*, *FYV6* and *XRS2*, encode three proteins required for the DNA repair process [25–27], has been identified in this study. The other six genes, *EAF1*, *ARP5*, *RSC1*, *RSC2*, *DCC1* and *CTF4* associated with chromatin modification, remodeling and cohesion [28–32], are all required for SDS tolerance. The *PHO85* gene, coding for a cyclin-dependent kinase Pho85, was screened in our study. The kinase Pho85 is involved in regulating the cellular responses of cell cycle progression, autophagy, response to DNA damage, phosphate and glycogen metabolism, establishment of cell polarity, as well calcium-mediated signaling. Therefore, deletion of the *PHO85* cause a decreased resistance to oxidative stress, chemicals, toxin, utilization of carbon and nitrogen [33–37]. In addition, we have identified two genes, *NEM1* and *CDC50*, which are required for normal nuclear envelope morphology and sporulation, or cell division, respectively [38, 39]. Taken together, these results suggests that SDS can affect the cell cycle and DNA processing of *S. cerevisiae* cells.

### Genes involved in aromatic amino acid biosynthesis and SDS tolerance

We have identified mutants for five genes involved in the synthesis of aromatic amino acids, *ARO1*, *ARO2*, *ARO7*, *TRP1* and *TRP5* that were sensitive to 0.03% SDS (Table 1; Fig. 1). The intracellular ROS levels in mutants for *ARO1*, *ARO7*, *TRP1* and *TRP5* were all higher than that of wide type cells when the cells were treated with SDS (Additional file 4: Fig. S3B and D). Previously, Aro1 catalyzes steps 2 through 6 in the biosynthesis of chorismate, which is a precursor to aromatic amino acids [23]; Aro2 catalyzes the conversion of 5-enolpyruvylshikimate 3-phosphate (EPSP) to form chorismate; and Aro7 catalyzes the conversion of chorismate to prephenate to initiate the tyrosine/phenylalanine-specific branch of aromatic amino acid biosynthesis [40–42]. Trp1 and Trp5 involved in the synthesis of tryptophan, where Trp1 catalyzes the third step in tryptophan biosynthesis and Trp5 catalyzes the last step of tryptophan biosynthesis [43, 44]. It was reported previously that *trp1–1* cells had a disadvantage in the response to SDS compared to auxotrophy for adenine, histidine, leucine or uracil when cells were grown on rich media [45]. They also showed that the cell membrane damage triggered by SDS was independent of CWI (cell wall integrity) signaling and was not a cause of tryptophan starvation. Our present results confirmed this previous findings that tryptophan exhibited protection from membrane disruptions and thus conferred resistance to SDS stress.

### SDS generates oxidative stress by regulating the expression of genes involved in redox homeostasis

**Fig. 3:**
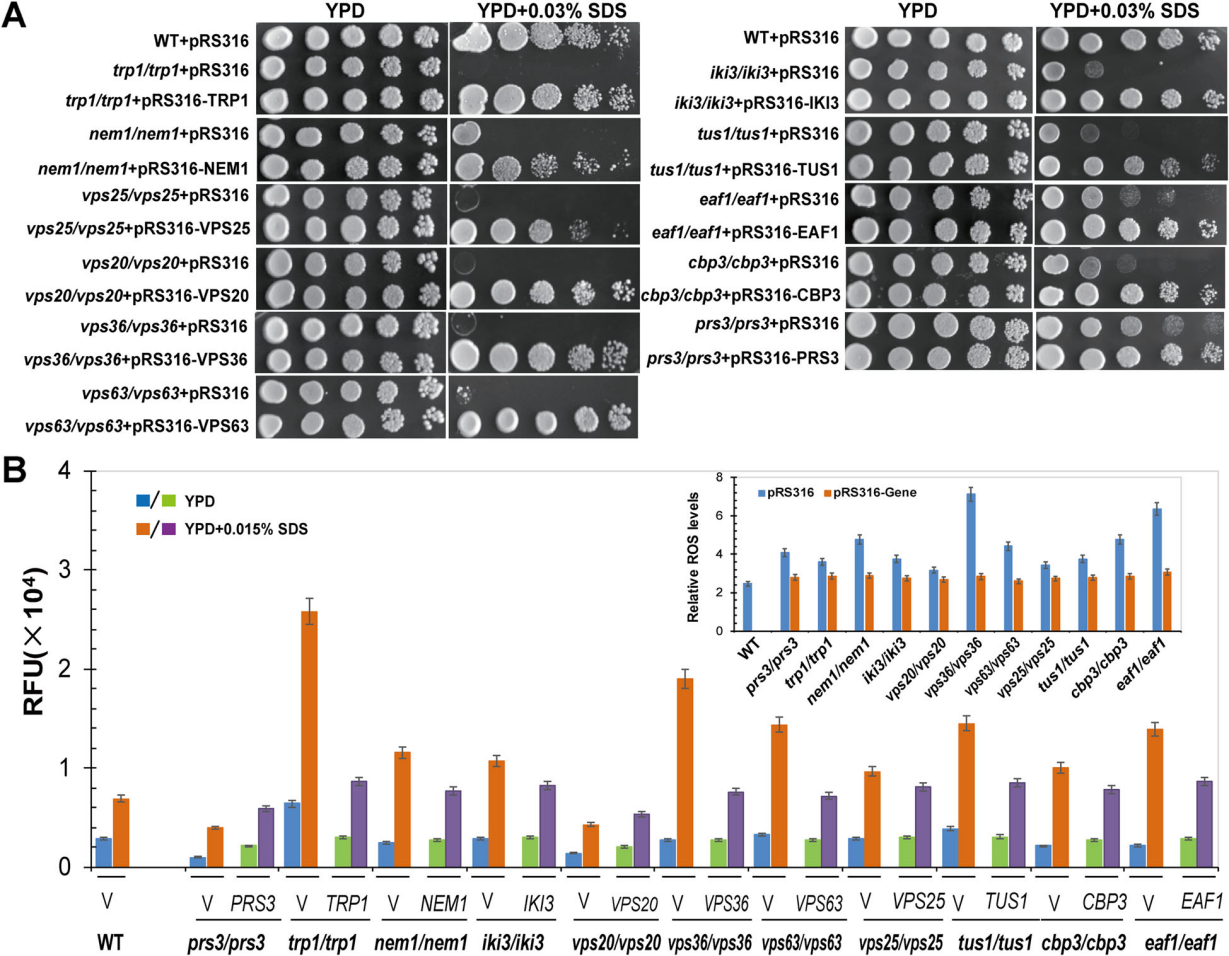
Introducing the conresponding genes back into the mutants surpress their SDS-sensitive and high intracellular ROS levels. **a** Complementation of the constructed expression plasmid in the sensitivity of the conresponding mutant to 0.03% SDS. **b** Intracellular ROS levels of the 11 indicated SDS-sensitive gene mutants in response to SDS stress. Strains containing the indicated plasmid were cultured in SD-URA over night for the complementary assay. To analyze the intracellular ROS levels, strains containing the indicated plasmid were first cultured to log-phase before being shifted to YPD with 0.015% SDS for additional 2 hours before they were collected for measurement of intracellular ROS levels stained by the dihydroethidium. The value is the average of three independent assays for each strain

The relative ROS levels in 11 mutants for *PRS3*, *TRP1*, *NEM1*, *EAF1*, *IKI3*, *CBP3*, *VPS20*, *VPS36*, *VPS63*, *VPS25*, and *TUS1* were all higher than that of wild-type cells (Fig. 2), indicating that these 11 genes were all important for dealing with the oxidative damage generated by SDS stress. To further confirm these results, we constructed the 11 plasmids expressing the above 11 genes in pRS316 plasmid, respectively, and then transformed them into the corresponding mutants. The growth defect of SDS-treatment mutant cells could be suppressed by introducing the expression plasmid back into the corresponding mutants (Fig. 3a), and their intracellular ROS levels were also recovered to that of the wild-type cells (Fig. 3b). Taken together, these results indicate that yeast cells lacking any of the above 11 genes are sensitive to SDS stress, leading to increased intracellular ROS levels.

**Fig. 4:**
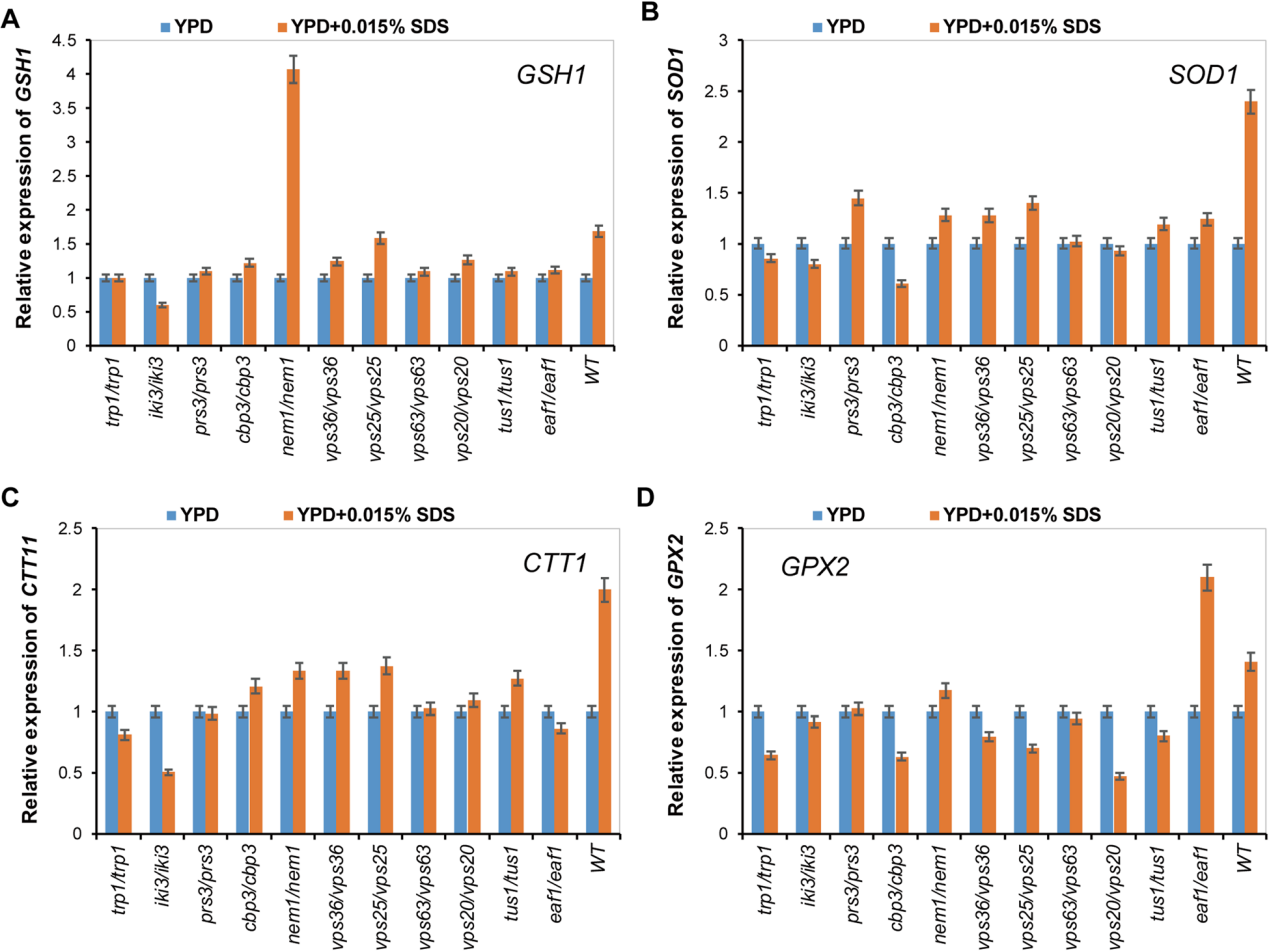
The expression of *GSH1*, *SOD1*, *CTT1* and *GPX2* genes coding for the antioxidant defenses are regulated by SDS stress. (**a-d**) WT and the indicated 11 mutants were treated in SDS medium for 1 h. The expression of the indicated genes was tested by qRT-PCR. The value is the average of three independent assays for each strain

It was reported that many of the oxidative stress scavenging genes could be induced by SDS stress in a DNA microarray analysis [2]. To investigate whether the deletion of genes *PRS3*, *TRP1*, *NEM1*, *EAF1*, *IKI3*, *CBP3*, *VPS20*, *VPS36*, *VPS63*, *VPS25*, and *TUS1* influence the expression of genes coding for the antioxidant defenses, we tested the expression of *GSH1* (glutamylcysteine synthetase), *SOD1* (cooper/zinc superoxide dismutase), *CTT1* (cytosolic catalase T), *GPX2* (2-Cys peroxiredoxin), *TRR1* (thioredoxin reductase) and *TRX2* (thioredoxin 2) by quantitative real-time PCR analyses. In the wild-type cells, the expression levels of *GSH1*, *SOD1*, *CTT1* and *GPX2* were significantly up-regulated after treatment with 0.015% SDS (Fig. 4), while no significant difference in the expression levels of *TRR1* or *TRX2* were observed when treated with or without SDS (Additional file 5: Fig. S4). Interestingly, both of the expression levels of *SOD1* and *CTT1* were reduced in the 11 mutants compared with wild type cells (Fig. 4b and c). In addition, the expression levels of *GSH1* and *GPX2* were also reduced in these mutants except the mutants for *NEM1* and *VPS25*, or *EAF1*, respectively (Fig. 4a and d). To investigate the decreased expression of *GSH1*, *SOD1*, *CTT1* and *GPX2*, we further analyzed the expression levels of these four genes in the wide type cells treated with 0.005 and 0.01% (Additional file 6: Fig. S5). We found that the expression levels of *GSH1*, *SOD1*, *CTT1* and *GPX2* were induced when the SDS concentrations were 0.01 and 0.015, but remain unchanged or slightly induced when the SDS concentration was 0.005%. It suggested that the expressions of the above four genes were dependent on the concentration of SDS. Overall, our results demonstrate that the decreased expression of *GSH1*, *SOD1*, *CTT1* and *GPX2* might be responsible for the high intracellular ROS levels accumulated in these mutants than wide type cells.

## Discussion

SDS is considered as a generally recognized safe ingredient for food and hygiene products. However, safety concern arises as oral ulcer or skin irritation was reported to be caused by products containing SDS in recent studies [46, 47]. *S. cerevisiae,* a budding yeast used in brewing beer and baking, is a single-celled eukaryote used extensively in laborary due to the fact that its genome has been sequenced and its genetics are easily manipulated. Here, we used *S. cerevisiae* to examiner the genome-wide SDS stress on eukaryotes and identified 108 SDS-sensitive mutants from the yeast nonessential gene deletion library, representing 2.3% of the screened 4757 gene deletion mutants. Previous study reported that 295 ORFs were up-regulated and 118 ORFs were down-regulated aftert SDS treatment, and the functional classifications of these genes were involved in a number of major cellular processes, including metabolism, protein sorting, transcription, cellular transport and biogenesis, DNA and protein synthesis, cellular communication / signal transduction and ionic homeostasis, etc. [2]. Interestingly, The 108 SDS-sensitive genes encoded proteins that are also involved in many of these cellular processes.

A significant aspect of SDS toxicity may be related to its effect on biological membranes that SDS may penetrate the membrane through pores in the yeast cell wall and destroy the membrane [48]. For example, SDS is used as a perturbing agent to cell wall integrity, and through *MPT5* and *SSD1* signaling pathway SDS can result in sensitivity to changes in external osmolarity, defect budding, and cell lysis [49]. Our results support this by showing that mutants for six genes (*TUS1*, *ROM2*, *SIT4*, *TOH1*, *SLG1* and *LAS21*) involved in the process of cell wall integrity were sensitive to SDS (Table 1 and Fig. 1). Interstingly, the intracellular ROS levels in these six mutants were all higher than wide type cells when cells were teated with SDS, indicating their crucial role in maintaining the cell wall integrity under SDS stress in *S. cerevisiae*.

Beside the stress on cell wall, SDS also introduced stresses on intracellular sorting and delivery of soluble vacuolar proteins. The largest functional category (28) of these identified SDS-sensitive genes are of cellular transport, transport facilities and transport routes. Vacuolar protein sorting (VPS) genes involved in vesicle transport to vacuoles play an important role in segregating molecules into distinct organelles and even affect the telomere length regulation [50]. Vacuolar H(+)-ATPase (V-ATPase) localized in the vacuole membrane (V-ATPase) is composed of the catalytic V1 subcomplex and the proton-translocating membrane V0 subcomplex [51]. It has a crucial role in the vacuolar system and acidification of the vacuole and other internal compartments including the whole secretory pathway [52]. The V1 complex is composed of at least eight subunits (A-H) encoded by eight *VMA* (Vacuolar Membrane ATPase) genes: *VMA1*, *VMA2*, *VMA4*, *VMA5*, *VMA7*, *VMA8*, *VMA10* and *VMA13*, respectively. The V0 complex is composed of at least five subunits (a, c, d, c’ and c”) encoded by six *VMA* genes: *VPH1*, *STV1*, *VMA3*, *VMA6*, *VMA11*, and *VMA16*, respectively. Three genes *VMA12*, *VMA21* and *VMA22* encode proteins that are required for the biogenesis of a functional V-ATPase [51]. Mutants for the VMA genes showed growth defects in response to oxidative stress, such as H_2_O_2_ [53]. In present study, 15 SDS-sensitive genes involved in the VPS pathway and four SDS-sensitive genes involved in V-ATPase function have been identified sensitive to SDS stress. We speculate that the absence of these genes might reduce the supply of cell wall and/or cell membrane components, leading to cell membrane damage or defects in cell wall structure in the SDS stress.

The expression of about 65 genes involved in the carbon metabolism were induced by SDS stress, including genes related to amino acid metabolism, C-compound and carbohydrate metabolism, lipid, fatty acid, and isoprenoid metabolism, vitamin, cofactor, and prosthetic group metabolism, and nucleotide metabolism [2, 54]. Mutants for a large group of 16 genes involved in metabolism is revealed to be sensitive to SDS stress in the present study, including six genes related to amino acids metabolism (*ARO1*, *ARO2*, *ARO7, TRP1, TRP5*, *PRS3* and *THR4*), four genes related to lipid and fatty acid metabolism (*ARG82*, *ELO3*, *IPK1,* and *ERG3*), four genes involved in nucleotide metabolism (*GRR1*, *REG1*, *ELM1* and *AFT1*), and one gene associated with carbohydrate metabolism (*NRK1*) (Table 1 and Fig. 1). In a previous study, it has been showed that the cell membrane damage trigged by SDS stress was independent of Cell Wall Integrity signaling pathway, and the biosynthesis of tryptophan and tyrosine played an important role in the SDS-induced plasma membrane stress response [45]. It is might explain why the six mutants for *ARO1*, *ARO2*, *ARO7, TRP1,*, *PRS3* and *TRP5,* involved in the synthesis of aromatic amino acids and tryptophan, were sensitive to SDS toxicity. In addition, lipid and fatty acid metabolism has significant role in maintaining the structures of cell membrane and cell wall, nucleotide metabolism is related to the processes of DNA synthesis, cell division and DNA repair, while carbohydrate metabolism is associated to cell growth and many other cellular activities. Moreover, it has been previously reported that, cell wall defects led to cells sensitive to SDS stress for a weakened cell wall allows it to penetrate more easily [8]. Therefore, it is not surprising that deletion mutants for the other ten genes involved in the above metabolism functions are sensitive to SDS stress.

Another concern with SDS toxicity has been its carcinogenicity; no evidence shows SDS-related tumorigenicity or carcinogenicity in early official review. SDS was extensively tested for genetic toxicity. Tests with SDS in bacterial or in mammalian systems (in vitro and in vivo) show no indication of genotoxicity with or without metabolic activation [55]. Published reports suggest that SDS has low acute mammalian toxicity and no known chronic effects. However, we have identified 14 SDS-sensitive genes of *S. cerevisiae* are involved in cell cycle and DNA processing in our study, though further investigation is required to clarify the significance.

Finally, we examined the intracellular ROS levels under SDS stress. Increased ROS level may result in significant damage to cell structures and constant high ROS level is known as oxidative stress. Most significantly, ROS is considered to damage DNA or RNA of cells. Under the SDS treatment, we observed most mutants (85/108) increased the intracellular ROS levels comparing with the wild-types, consistent with their being-affected growth. We pick up 11 mutants for *PRS3*, *TRP1*, *NEM1*, *EAF1*, *IKI3*, *CBP3*, *VPS20*, *VPS36*, *VPS63*, *VPS25*, and *TUS1*, which accumulated higher relative ROS levels than that of wild-type cells under SDS treatment (Fig. 2), to investigate the mechanism of oxidative damage induced by SDS stress. We have shown that the expression of some antioxidant defenses genes were down-regulated by SDS stress in these mutants. It suggests that some of the SDS-sensitive genes might be involved in maintaining the redox balance under SDS treatment. However, some mutants reduced its ROS production, the genes involved in these mutants may be related the detoxification of SDS by yeast cells. Another interesting result of our study is that six mutants for *ARG82*, *TRP5*, *GRR1*, *MSH1*, *LAS21*, and *YNL296W*, accumulated lower intracellular ROS levels under 0.015% SDS treatment when compared with no SDS treatment. They could also play a role in detoxification of SDS by yeast cells, but further investigations are needed.

## Conclusions

To study the SDS toxicity we have performed a genome-wide screen for mutants that are sensitive to 0.03% SDS. It is demonstrated that the intracellular ROS levels in 85 of the identified 108 SDS-sensitive mutants were significantly higher than that of the wild-type BY4743 cells in response to SDS stress. In addition, the expression levels of genes involved in antioxidant defenses suggest that SDS might generate oxidative damage by regulating these genes, leading to cells sensitive to SDS stress. Taken together, our present study provides a potential way to understand the detoxification mechanisms of SDS by yeast cells.

## Methods

### Strains, media and culture conditions

All *S. cerevisiae* strains were derived from the S288C genetic background. The homozygous diploid deletion mutant library were purchased from Invitrogen Inc. [http://clones.invitrogen.com/] and was frozen at − 80 °C in 96-well microtitre plates in 15% glycerol in liquid YPD medium (1% yeast extract, 2% peptone, 2% glucose). SD-URA media (0.17% (w/v) yeast nitrogen base, 2% (w/v) glucose, 0.5% ammonia sulfate, adding 1/10 mL amino acid mixture without uracil) was used to culture yeast cells for plasmid selection. Solid media were produced by adding 2% (w/v) agar when necessary. SDS was purchased from Sangon Biotech (Shanghai, China), and Dihydroethidium was purchased from Sigma (Beijing, China).

### Primary screen for mutants involved in SDS toxicity

Prior to the primary screen for SDS-sensitive mutations, the deletion mutant library was first transferred to fresh liquid YPD medium and cultured at 30 °C. Then each mutant strains were transferred to fresh liquid YPD medium with or without 0.015% SDS, respectively, and cultured at 30 °C for about 6 to 12 h. The growth rates of each mutant in YPD medium with and without 0.015% SDS were measured at OD_600_ to determine the SDS-sensitivity in the primary screen. The SDS sensitive strains showed reduced growth and was defined as mutants with a relative OD_600_ reduced by more than 30% in liquid YPD medium containing supplemented SDS but not in liquid YPD medium without supplemented SDS as compared to that of the wild-type.

### Phenotypic analysis by spot dilution growth assays

The identified mutants that appeared sensitive were subjected to the secondary screen retested by spot dilution growth assays. In brief, each mutants were cultured overnight in liquid YPD at 30 °C and then were spotted onto YPD plates with or without 0.03% SDS in comparison to the wild type BY4743 strain.

To further confirm the SDS sensitivity of mutants for *PRS3*, *TRP1*, *NEM1*, *EAF1*, *IKI3*, *CBP3*, *VPS20*, *VPS36*, *VPS63*, *VPS25*, and *TUS1* genes, we introduced the pRS316 vector and pRS316 vector expressing the conresponding genes back into mutants for *PRS3*, *TRP1*, *NEM1*, *EAF1*, *IKI3*, *CBP3*, *VPS20*, *VPS36*, *VPS63*, *VPS25*, and *TUS1*. The sensitivity to SDS of the transformants was examined on YPD and YPD + 0.03% SDS plates using the above serial dilution assay method.

### DNA manipulations

To express the *TRP1* gene in the plasmid pRS316, the DNA fragment which contains the promoter, ORF and terminator region, was first amplified with primers TRP1-F and TRP1-R (Additional file 1: Table S1), and were cloned into the *Bam*HI and *Hin*dIII sites of pRS316 to yield pRS316-*TRP1*. The other plasmids of pRS316- *IKI3*, pRS316-*PRS3*, pRS316-*CBP3*, pRS316-*NEM1*, pRS316-*VPS36*, pRS316-*VPS25*, pRS316-*VPS63*, pRS316-*VPS20*, pRS316-*TUS1* and pRS316-*EAF1* were all constructed by the same method described above. All the inserts were confirmed by DNA sequencing.

### Oxidative stress assay for SDS-sensitive mutants

To determine the cellular oxidative stress of the SDS-sensitive mutants, we tested the intracellular ROS level by the dihydroethidium as previously described [56]. Briefly, overnight cell cultures were inoculated in YPD to an optical density OD_600_ = 0.1, grown to middle log phase, and split into two aliquots with or without 0.015% SDS and were grown for 2 h. Then about 5 × 10^6^ cells were harvested by centrifugation and resuspended in 250 μl PBS with 2.5 μg/ml DHE, and incubated in the dark for 30 min. The relative fluorescence units (RFU) were tested by a fluorescence reader (Synergy™ H4, BioTek).

### RNA extraction and quantitative PCR analysis

The mutants were first grown to middle log phase (OD_600_ = 0.6–1.0), and then they were grown in the presence or absence of 0.015% SDS for 1 h. The total RNA was extracted by hot phenol method. The genomic DNA was first removed from the total RNA with RNase-free DNase I. The first-strand cDNA synthesis was performed using the Primer Script RT reagent kit (Cwbiotech, China) according to the manufacturer’s instructions. The expression mRNA levels of *TRR1*, *TRX2*, *GSH1*, *SOD1*, *CTT1* and *GPX2* were detected by quantitative PCR (qPCR) as described previously [57] (Additional file 1: Table S1). Each reaction was carried out in triplicate.

### Enrichment analysis for the identified genes

The web-based tool (http://metascape.org/gp/index.html#/main/step1) was used for enrichment analysis of SDS-sensitive genes. *p*-value < 0.01, min overlap genes = 3, and min enrichment factor > 1.5 were set as the cutoff criteria and the significance was ranked by enrichment score (−log 10 (*P*-value)).

## Supplementary information

**Additional file 1 **: **Table S1.** Primers used in this study.

**Additional file 2 **: **Fig. S1.** Genotype confirmation of the 108 gene deletion mutants by PCR. Cells of the 108 gene mutants were grown overnight in YPD medium at 30 °C and then collected for DNA extraction. PCR was performed with genomic DNA of each of these mutants with the primer located at the upstream of its open reading frame and the reverse primer KanMX4-R from the internal sequence of the KanMX4. PCR products were separated on 1% agarose gel, and sizes of the DNA marker were indicated on the left or right of the gel.

**Additional file 3 **: **Fig. S2.** Meta-enrichment analysis summary of SDS-sensitive genes. Heatmap of the top 16 enriched GO terms. For GO terms, each band represents one enriched term coloured according to its -log 10 *p*-value. The dominant term within each group is used as a group heading.

**Additional file 4 **: **Fig. S3.** Intracellular ROS levels of 108 SDS-sensitive gene mutants in response to SDS stress. a: Metabolism; b: Cell cycle and DNA Processing; c: Transcription; d: Protein with Binding Function or Cofactor Requirement (structural or catalytic); e: Cellular Transport, Transport Facilities and Transport Routes; f: Biogenesis of cellular components; g: Cell wall integrity and osmotic stress response; h: Unclassified Proteins. Log-phase cells were grown with or without 0.015% SDS for two hours before they were collected for measurement of intracellular ROS levels stained by the dihydroethidium. The intracellular ROS levels of these SDS-sensitive mutants were listed according to their categories in comparison to that of wild type cell BY4743. The value is the average of three independent assays for each strain.

**Additional file 5 **: **Fig. S4.** The expression of *TRR1* and *TRX2* under SDS stress. (A-B) WT and the indicated 11 mutants were treated to SDS medium for 1 h. The expression of the indicated genes was tested by qRT-PCR. The value is the average of three independent assays for each strain.

**Additional file 6 **: **Fig. S5.** The expression levels of *GSH1*, *SOD1*, *CTT1* and *GPX2* genes in response to different concentrations of SDS in the wide type BY4743 cells. The expression of the indicated genes was tested by qRT-PCR. The value is the average of three independent assays for each strain.

## Data Availability

All data generated or analysed during this study are included in this published article and its supplementary information files.

## Abbreviations

CWI: Cell wall integrity
QPCR: Quantitative PCR
RFU: Relative fluorescence units
ROS: Reavtive oxygen species
SDS: Sodium dodecyl sulfate
VMA: Vacuolar membrane ATPase
VPS: Vacuolar protein sorting
