# Supplementary material for: Genome-wide identification for genes involved in sodium dodecyl sulfate toxicity in Saccharomyces cerevisiae
Source: BMC Microbiol. 2020 Feb 17;20:34. doi: 10.1186/s12866-020-1721-2 (PMC7027087; doi:10.1186/s12866-020-1721-2)
Supplement: Supplementary file 1 — Additional file 1 : Table S1. Primers used in this study. [file 12866_2020_1721_MOESM1_ESM.docx]

**Table S1.** Primers used in this study

| Name | Sequence (5’-3’) |
| --- | --- |
| TRR1-F | TATGATGGCGAACGGTATTGCTG |
| TRR1-R | ACAGGTTCTGCGTCTTCGTTAAATTC |
| TRX2-F | ATGGTCACTCAATTAAAATCCGCTTC |
| TRX2-R | TTGTAGAAGATTAGGGTAGGCATGGAAG |
| GSH1-F | TCCATATTTGAATTACGTGGGTAGTTACG |
| GSH1-R | AAGCGGCATTTTTATGATTCCACG |
| SOD1-F | TGTTAAAGGGTGATGCCGGTGTCTC |
| SOD1-R | TTCGTCAGTTGGAGCACCATGTGTC |
| CTT1-F | TTCCTCAGAGACGCTATTAAGTTTCCC |
| CTT1-R | TCTTTGTTGACCATGATGAAGGAATGAC |
| GPX2-F | AATGCAAGGACAAGAAAGGCGAATC |
| GPX2-R | TTCCTGCTTCCCGAACTGATTACATG |
| PGK1-RT-F | TACGTTGTCTTGGCTTCTCACTTGG |
| PGK1-RT-R | TTGGAAGCCTTGACCTTTTGACC |
| TRP1-F | GCCGCTCTAGAACTAGT***GGATCC***TTGAAGGAACCACTGACGAAGG, *Bam*HI site underlined |
| TRP1-R | AGGTCGACGGTATCGAT***AAGCTT***ATCGGGTCATTGTAGCGTATG, *Hind*Ⅲ site underlined |
| IKI3-F | GCCGCTCTAGAACTAGT***GGATCC***TTCCAGTTCCTGTTGTAGCCTG, *Bam*HI site underlined |
| IKI3-R | AGGTCGACGGTATCGAT***AAGCTT***AAGGATATTGCCAGAACTCAAAC, *Hind*Ⅲ site underlined |
| PRS3-F | GCCGCTCTAGAACTAGT***GGATCC***ATCGAAGGACTACAAGCGAAC, *Bam*HI site underlined |
| PRS3-R | AGGTCGACGGTATCGAT***AAGCTT***ATGGGAAATGTGTTTTTTGGTC, *Hind*Ⅲ site underlined |
| CBP3-F | GCCGCTCTAGAACTAGT***GGATCC***ACGAGTTCACGTTTCCTGAAC, *Bam*HI site underlined |
| CBP3-R | AGGTCGACGGTATCGAT***AAGCTT***CTTCCTTTATCGGTGGGTTAG, *Hind*Ⅲ site underlined |
| NEM1-F | GCCGCTCTAGAACTAGT***GGATCC***ATACGACCAGATGTTGTTTGAGG, *Bam*HI site underlined |
| NEM1-R | AGGTCGACGGTATCGAT***AAGCTT***TTTAGCGTGTCACTGCTGAAG, *Hind*Ⅲ site underlined |
| VPS36-F | GCCGCTCTAGAACTAGT***GGATCC***ACATGGAAGAGCAACATTCTCG, *Bam*HI site underlined |
| VPS36-R | AGGTCGACGGTATCGAT***AAGCTT***AAGGATGTGGAAGTCGTGCG, *Hind*Ⅲ site underlined |
| VPS25-F | GCCGCTCTAGAACTAGT***GGATCC***TGGAACGTAGCACATTTTACTG, *Bam*HI site underlined |
| VPS25-R | AGGTCGACGGTATCGAT***AAGCTT***AAGTTGAATGGTAAGGTTCAAGAC, *Hind*Ⅲ site underlined |
| VPS63-F | GCCGCTCTAGAACTAGT***GGATCC***TGCTTCTATTCGTTAGTGCTGG, *Bam*HI site underlined |
| VPS63-R | AGGTCGACGGTATCGAT***AAGCTT***ACAGATTTCGATTCTATGTGACAC, *Hind*Ⅲ site underlined |
| VPS20-F | GCCGCTCTAGAACTAGT***GGATCC***AGGCATATAGACCAAGTGACTGAG, *Bam*HI site underlined |
| VPS20-R | AGGTCGACGGTATCGAT***AAGCTT***AGTTGCCATAATTGGGACAG, *Hind*Ⅲ site underlined |
| TUS1-F | GCCGCTCTAGAACTAGT***GGATCC***ACCCGATCTTGGTATGTATGC, *Bam*HI site underlined |
| TUS1-R | AGGTCGACGGTATCGAT***AAGCTT***ACCAGAAGTGGATAGCGCAG, *Hind*Ⅲ site underlined |
| EAF1-F | GCCGCTCTAGAACTAGT***GGATCC***AAATATGCCAAGATTCACTTCG, *Bam*HI site underlined |
| EAF1-R | AGGTCGACGGTATCGAT***AAGCTT***AGATGGTATCAAACAGATTGCTTC, *Hind*Ⅲ site underlined |
| ARO2-F | AGACTTGCCTGCGGTAGTCTTTG |
| MET7-F | AAGACGCAGAACCCGTTAC |
| TRP5-F | TCCAAATAGCACCGCTACAATGC |
| ARO7-F | TGTACCACCCTGACCATTCCG |
| THR4-F | TGCCGAAGACAGCAGTAACG |
| AGR82-F | GGAGCATTTGGATCACGTTCG |
| ELO3-F | TGGCAATCTATCGTGTGGG |
| ROM2-F | TGATCTTGTACTGGAACTTGG |
| IPK1-F | TGATGCGTGACATATCAAGGTAC |
| NRK1-F | TGGGTGCCATCCATAGTGG |
| TRP1-F | TGCATTGAGGCTCTGTTTGG |
| GRR1-F | TCGTTGCCACAACTGAGCC |
| REG1-F | TGGCCTCAAATTGTTCTTTGGAAC |
| AYT1-F | TCGGTGGATGACAATAGTTTG |
| ERG3-F | ACTCAGCATCTCTTCGTCATCC |
| PRS3-F | AAGATGGCGAGCTGGCGGC |
| ARO1-F | AGGCTCGACAGAGGAATATC |
| SLX5-F | TATTGATTCTACTGTAGACGCGG |
| SLX8-F | TCGCTATCTGGATGTAGGC |
| ARP5-F | TGGATGATGTCCTGAATGG |
| RSC2-F | TTGGCTTCCGCTTCATTG |
| PHO85-F | ACTCTCAGATGTTTCACACGAC |
| SIT4-F | TGCGGCCACGTCGTGCC |
| FSC1-F | TACTGGCAACGTAGACAAGG |
| FYV6-F | TCTGTGCGAAGTGAATCC |
| DCC1-F | TGGACTCAAATTCTCAATGG |
| EAF1-F | ATGTTGCCACGGCACTTGG |
| XRS2-F | TCTCAAATGTCCAAAGAGGGC |
| NEM1-F | TTAACATCGGCTCGTCC |
| CTF4-F | AGGGTGAGGAACCCACTCTAACC |
| BRE1-F | TTCCATGTCTCTGGATGAATG |
| MOT2-F | TACCACGGAGGATGGTG |
| SPT4-F | ACATGACTGCGTATAGTGGTTC |
| CTK1-F | TTACTACCATCAGCAAGTCTGG |
| POP2-F | TGGTGAAACTAAGGTCTGGC |
| BDF1-F | ACATAAGAGCAGCTCTTATGC |
| SAC3-F | AGCAGAAGACAAGACACACG |
| RPL7A-F | ACCTTGGCCTTTGCACCGTG |
| IKI3-F | AACTCCAAGGCGGCAGCC |
| DEG1-F | ACCCGTCCTTCTTTGTTTAG |
| SFL1-F | ACCGCCAGTTCTTTGGAG |
| SRB5-F | ACCTCGGATGCCTACG |
| DEP1-F | TAGACTGAGACAGTGCGCCC |
| LSM1-F | ACGGCAAGGTAACAGACGG |
| VPS64-F | TAAGCGCCAAATTGAACAG |
| OTC1-F | TCCAAACCTGACTACCAAATG |
| VID22-F | TAAGTCATTGTTCCTGGCG |
| VMA21-F | TGCCTGCTTCTTCTATTTGC |
| SGF29-F | TCGTGTGCAACACCGAGGTC |
| LAS21-F | TAGCAGCGCATCCCTATGCG |
| DIA4-F | ACGCAGGCGACCAGGGC |
| CBP3-F | ATGGACCGTATTGAAGTATC |
| VPS1-F | TTCATCCAGTTCCTTCCTCTC |
| MAP1-F | TTCGTCAATGAACCTTCTCC |
| APL2-F | AGCAAAGATGGCTATGCTGG |
| RPL35A-F | TCCGGTGAAGGTTTGTATG |
| NUP84-F | ATGGAACTCGCACTGACCC |
| RPL13B-F | ACTATGCGTACTCATTTACTGCC |
| VPS33-F | TGCTCCAAGACATTAACCGC |
| PHO87-F | AGACTATCTCTCCCGATAGATG |
| ISA1-F | TCCAAGTATGCTTACGCCGC |
| PIL1-F | AGACCTGGGATCACCTCGC |
| VPS36-F | TCCTCATGCTTATAGCGTCC |
| SNF8-F | TGCTCCAAGGTAGTGCC |
| VMA5-F | TCATGTCCAACAACTTGGTC |
| CHS5-F | TGCGCGAGATTGTAGGTTC |
| VPS38-F | TAGTGACGCCGTTCAAACTGC |
| VPS24-F | AGCTGAAGCGTGTCGAAG |
| MIP1-F | TTGGAGACCACAACACCGC |
| RIC1-F | TACACTTGCCAGGTCCGC |
| RCY1-F | AGATTTCTGTGTCTTCAGGG |
| VPS51-F | AGCCTTCTACCGCTACTAAAG |
| VPS20-F | TCAGAAGGATTTGATGTAAGG |
| VMA3-F | AATGTCCAGTGAGAGGATG |
| STP22-F | TCCTGTTTCTGCCTCATGTC |
| SNF7-F | ACAACGTGATAAGTGATGGTG |
| SRN2-F | TATCGCGTCTTACGGATG |
| YPT31-F | ACTGGTCTCAACCTCGGTTGC |
| VPS25-F | AGAGGGAACGTGTGGGC |
| VPS16-F | ATGCGGCATATCTGCACATAG |
| GOS1-F | TGGGTTCCTTTGCTTGGAC |
| LOA1-F | AACGGATCATCGACCATACTC |
| VMA13-F | ACGGTGCCGGTTGTCACAC |
| HIT1-F | ATCAATCCTTGCATTTCAGC |
| CDC50-F | AGAACTTCTTCAAATGCGG |
| MRP124-F | TGTATATCAGCTACTGCTCCC |
| MDM10-F | ATCTGTGTATTTCTCGTCCC |
| MDM20-F | ATGATATGTCTTCTTCGCC |
| SLG1-F | TCTCTTCCAGACTTCGGTTTAC |
| GIM5-F | AGGGTATCTTCAGAGTTGTTATC |
| MRPL32-F | ACCTTTCACGTTGGGCTGTC |
| PBS2-F | ACTTCAACAGCTACGGCC |
| TUS1-F | ATGGAACGGCTTTACCCGATC |
| API2-F | AACCAACATCGGACGAG |
| YDLO41W-F | AGCATTGAGAGACTCTCGTTTAG |
| YLR358C-F | AGAAGACAATGGCGTAGTG |
| YOR149C-F | TAGCGTGTTTGGTACGTGC |
| YPR123C-F | TCTTTGGAGTTTGCTGAAGG |
| YGR272C-F | ATCACGGCTTTCTCGG |
| YLR374C-F | AACAGATGAAGGACCAGCC |
| YNL296W-F | ACAGATTCAATGGAAAGCC |
| YKL136W-F | ACGAGATGAAATGAAATCGTGCC |
| TOH1-F | TACGGTCTTTGCTTGGC |
| YDR008C-F | ATGATTACGAGGATACGGAG |
| SRF1-F | ACGGGTGTGGTAACCG |
| YGR160W-F | AGCGTCTCTGTCAGCATTG |
| YOR331C-F | TGGAGATAACAATCTCTTCC |
| BRP1-F | ACTTTCTGTGAACGAGGG |
| BUD30-F | TCCATTCTTCGTCATCC |
| KANMX4-R | GCCGTTTCTGTAATGAAGGAG |
