# Supplementary figures and images for: Genome-wide identification for genes involved in sodium dodecyl sulfate toxicity in Saccharomyces cerevisiae
Source: BMC Microbiol. 2020 Feb 17;20:34. doi: 10.1186/s12866-020-1721-2 (PMC7027087; doi:10.1186/s12866-020-1721-2)

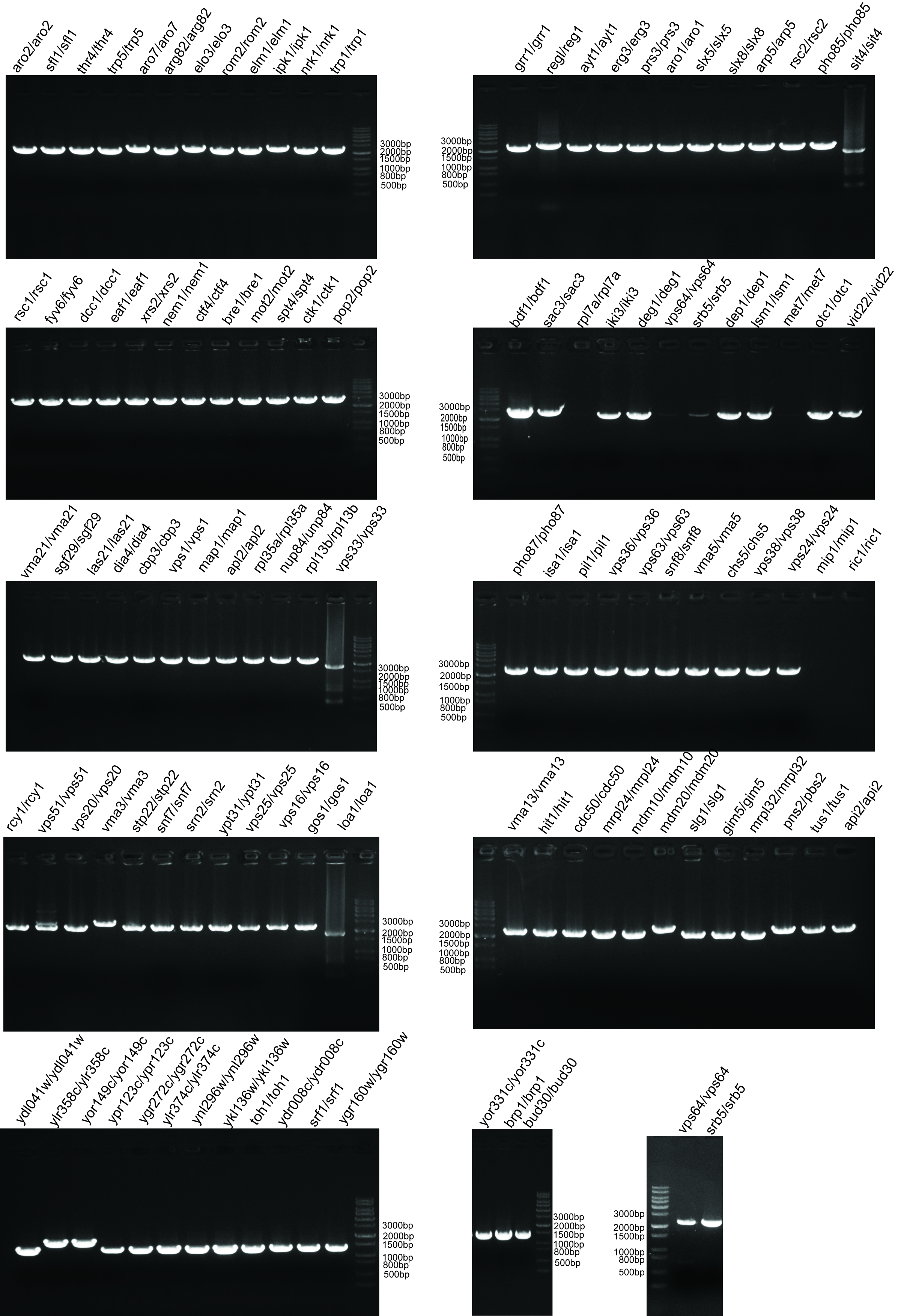

Supplement: Supplementary file 2 — Additional file 2 : Fig. S1. Genotype confirmation of the 108 gene deletion mutants by PCR. Cells of the 108 gene mutants were grown overnight in YPD medium at 30 °C and then collected for DNA extraction. PCR was performed with genomic DNA of each of these mutants with the primer located at the upstream of its open reading frame and the reverse primer KanMX4-R from the internal sequence of the KanMX4. PCR products were separated on 1% agarose gel, and sizes of the DNA marker were indicated on the left or right of the gel. [file 12866_2020_1721_MOESM2_ESM.tif]

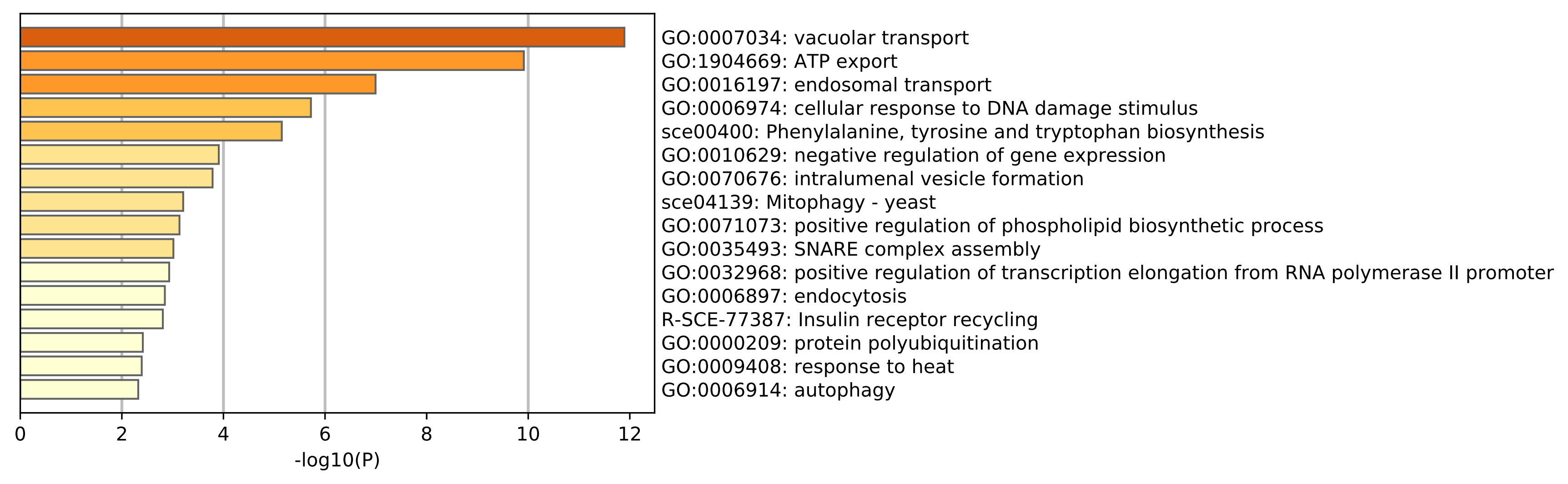

Supplement: Supplementary file 3 — Additional file 3 : Fig. S2. Meta-enrichment analysis summary of SDS-sensitive genes. Heatmap of the top 16 enriched GO terms. For GO terms, each band represents one enriched term coloured according to its -log 10 p-value. The dominant term within each group is used as a group heading. [file 12866_2020_1721_MOESM3_ESM.tif]

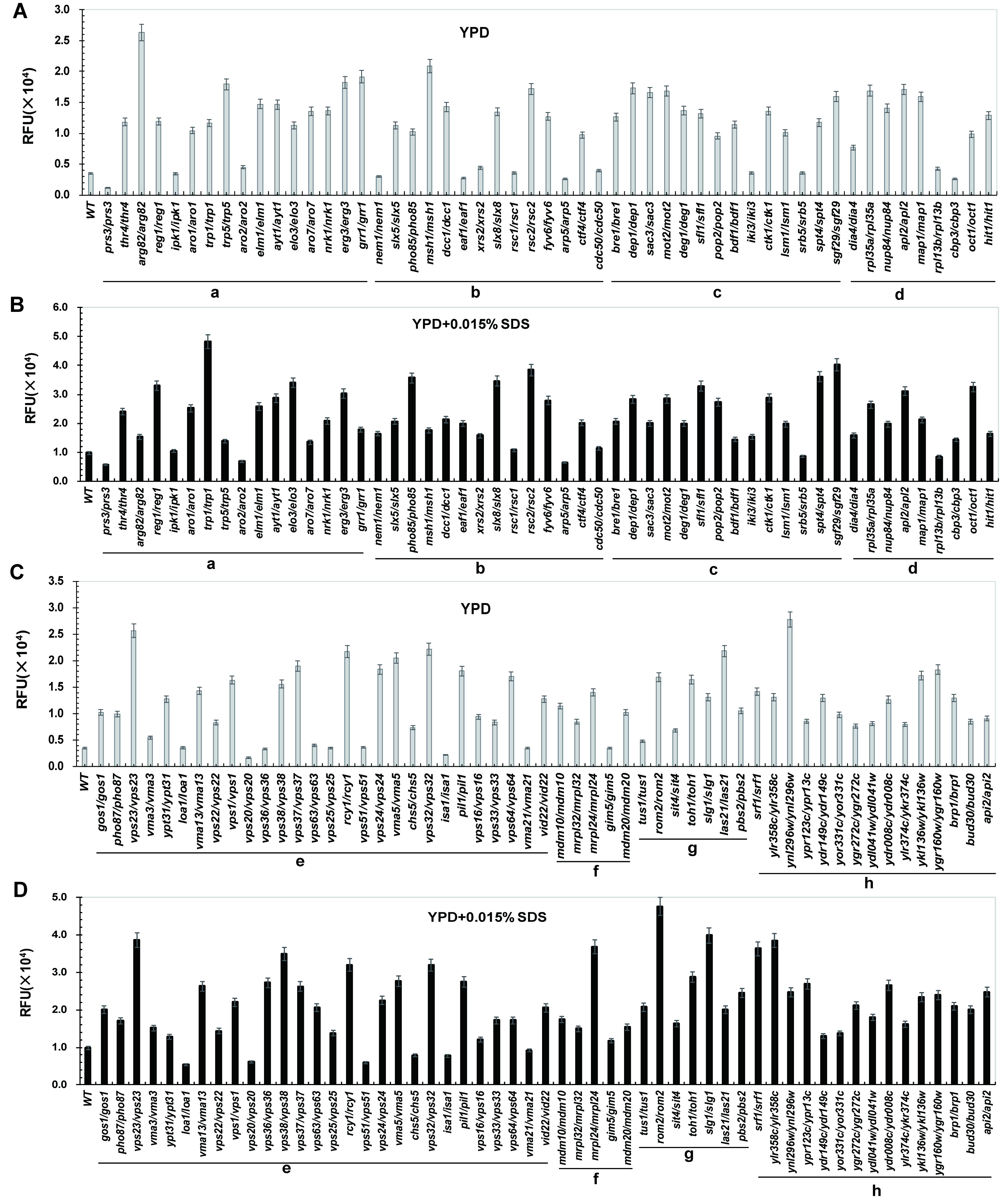

Supplement: Supplementary file 4 — Additional file 4 : Fig. S3. Intracellular ROS levels of 108 SDS-sensitive gene mutants in response to SDS stress. a: Metabolism; b: Cell cycle and DNA Processing; c: Transcription; d: Protein with Binding Function or Cofactor Requirement (structural or catalytic); e: Cellular Transport, Transport Facilities and Transport Routes; f: Biogenesis of cellular components; g: Cell wall integrity and osmotic stress response; h: Unclassified Proteins. Log-phase cells were grown with or without 0.015% SDS for two hours before they were collected for measurement of intracellular ROS levels stained by the dihydroethidium. The intracellular ROS levels of these SDS-sensitive mutants were listed according to their categories in comparison to that of wild type cell BY4743. The value is the average of three independent assays for each strain. [file 12866_2020_1721_MOESM4_ESM.tif]

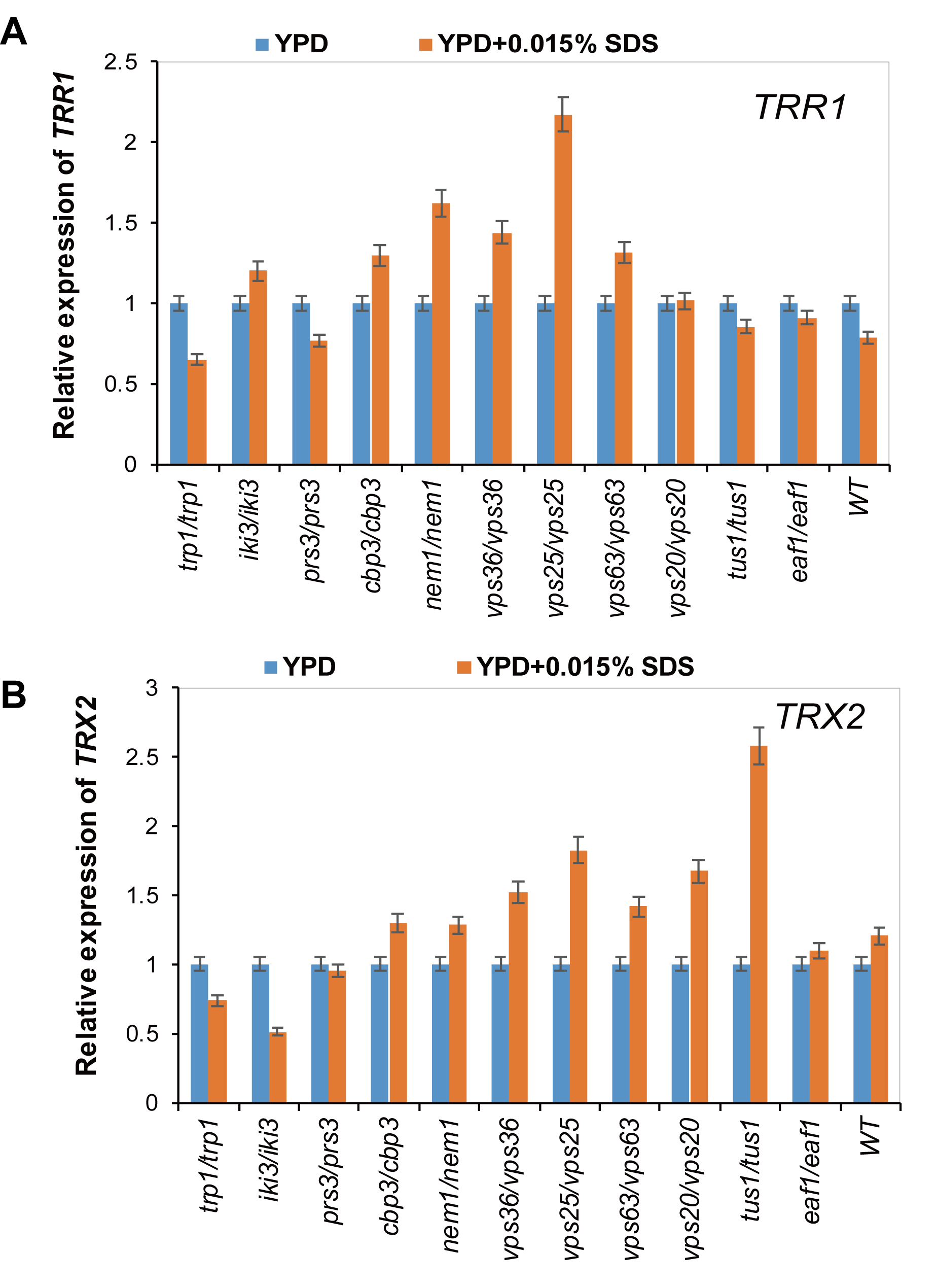

Supplement: Supplementary file 5 — Additional file 5 : Fig. S4. The expression of TRR1 and TRX2 under SDS stress. (A-B) WT and the indicated 11 mutants were treated to SDS medium for 1 h. The expression of the indicated genes was tested by qRT-PCR. The value is the average of three independent assays for each strain. [file 12866_2020_1721_MOESM5_ESM.tif]

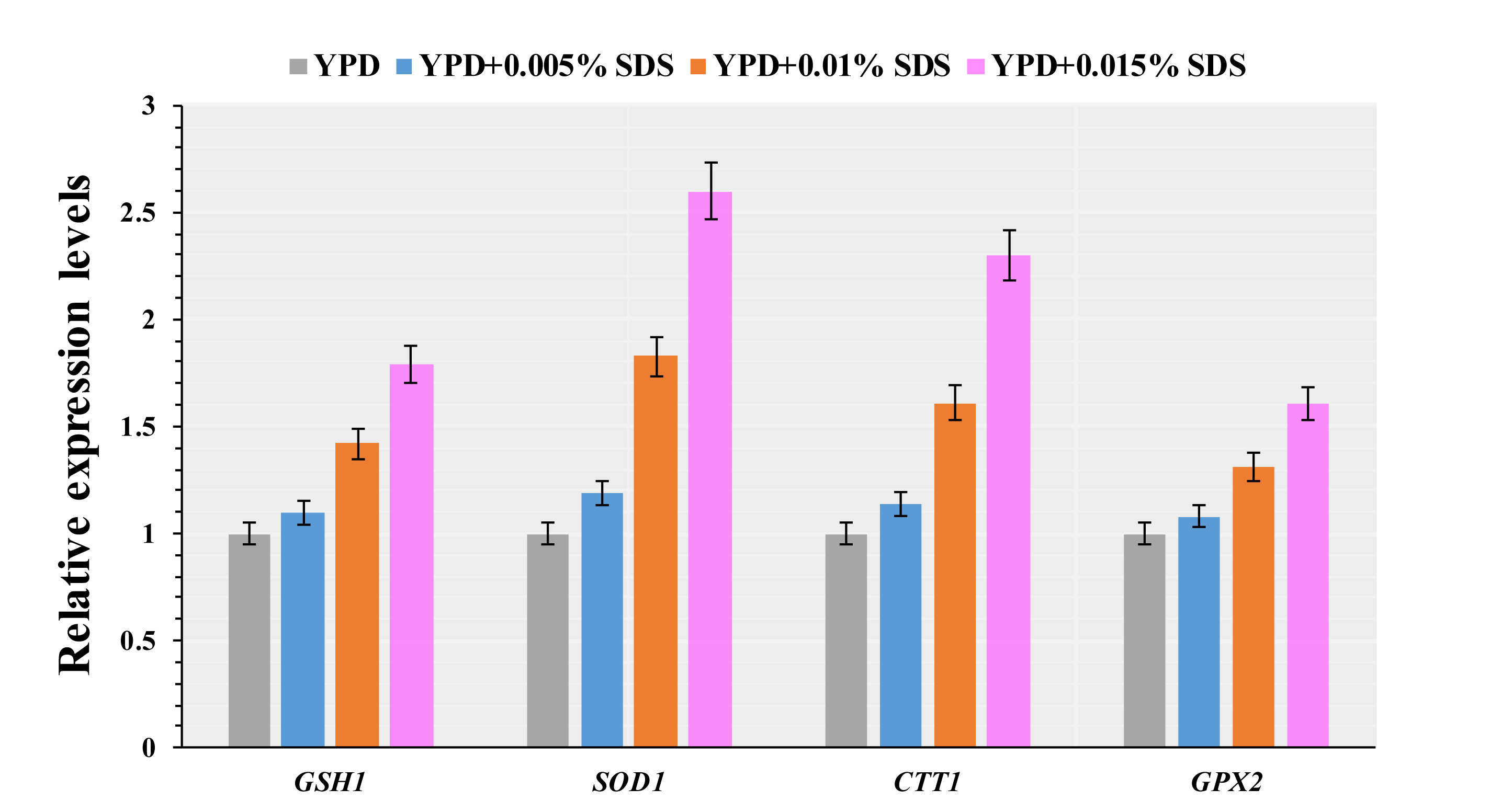

Supplement: Supplementary file 6 — Additional file 6 : Fig. S5. The expression levels of GSH1, SOD1, CTT1 and GPX2 genes in response to different concentrations of SDS in the wide type BY4743 cells. The expression of the indicated genes was tested by qRT-PCR. The value is the average of three independent assays for each strain. [file 12866_2020_1721_MOESM6_ESM.tif]
